# Comparative genomic analysis of *Proteus* spp. isolated from tree shrews indicated unexpectedly high genetic diversity

**DOI:** 10.1371/journal.pone.0229125

**Published:** 2020-02-21

**Authors:** Wenpeng Gu, Wenguang Wang, Pinfen Tong, Chenxiu Liu, Jie Jia, Caixia Lu, Yuanyuan Han, Xiaomei Sun, Dexuan Kuang, Na Li, Jiejie Dai

**Affiliations:** 1 Center of Tree Shrew Germplasm Resources, Institute of Medical Biology, Chinese Academy of Medical Sciences and Peking Union Medical College, Yunnan Key Laboratory of Vaccine Research and Development on Severe Infectious Diseases, Yunnan Innovation Team of Standardization and Application Research in Tree Shrew, Kunming, China; 2 Department of Acute Infectious Diseases Control and Prevention, Yunnan Provincial Center for Disease Control and Prevention, Kunming, China; University of Minnesota, UNITED STATES

## Abstract

*Proteus* spp. are commensal gastrointestinal bacteria in many hosts, but information regarding the mutual relationships between these bacteria and their hosts is limited. The tree shrew is an alternative laboratory animal widely used for human disease research. However, little is known about the relationship between *Proteus* spp. and tree shrews. In this study, the complete genome sequencing method was used to analyse the characteristics of *Proteus* spp. isolated from tree shrews, and comparative genomic analysis was performed to reveal their relationships. The results showed that 36 *Proteus* spp. bacteria were isolated, including 34 *Proteus mirabilis* strains and two *Proteus vulgaris* strains. The effective rate of sequencing was 93.53%±2.73%, with an average GC content of 39.94%±0.25%. Briefly, 3682.89±90.37, 2771.36±36.01 and 2832.06±42.49 genes were annotated in the NCBI non-redundant nucleotide database (NR), SwissProt database and KEGG database, respectively. The high proportions of macrolide-, vancomycin-, bacitracin-, and tetracycline-resistance profiles of the strains were annotated in the Antibiotic Resistance Genes Database (ARDB). Flagella, lipooligosaccharides, type 1 fimbriae and P fimbriae were the most abundantly annotated virulence factors in the Virulence Factor Database (VFDB). SNP variants indicated high proportions of base transitions (Ts), homozygous mutations (Hom) and non-synonymous mutations (Non-Syn) in *Proteus* spp. (P<0.05). Phylogenetic analysis of *Proteus* spp. and other references revealed high genetic diversity for strains isolated from tree shrews, and host specificity of *Proteus* spp. bacteria was not found. Overall, this study provided important information on characteristics of genome for *Proteus* spp. isolated from tree shrews.

## Introduction

*Proteus* spp. are common commensal gastrointestinal bacteria in many hosts and belong to the *Morganellaceae* family [1–2]. Currently, the genus comprises *Proteus mirabilis*, *Proteus vulgaris*, *Proteus penneri*, *Proteus hauseri*, and unnamed genomospecies 4, 5, and 6 [3–4]. These bacteria are considered as opportunistic human pathogens and have been isolated from different clinical sources, such as urine and wounds [5]. Many studies have demonstrated their roles in the pathogenesis of infections in human beings, indicating that their virulence factors enable the bacteria to reach and survive different niches of host organisms. However, *Proteus* spp. is actually a normal, ubiquitous commensal constituent of human flora and it is recognized that the gut is a reservoir for this proteolytic organism. Previous studies have illustrated the *Proteus* spp. antagonistic or commensal relationships with numerous animals and sometimes found cooperated relations with different animal hosts, acting as animal symbionts [5–7]. However, the complicated interdependence between *Proteus* spp. and animals coexisting with the bacteria in the environment is still unclear. Information regarding the mutual relationships between *Proteus* spp. bacteria and their natural habitats is currently limited.

The tree shrew (*Tupaia belangeri*) is a new laboratory animal belonging to the family Tupaiidae, order Scandentia, widely distributed in South Asia, Southeast Asia and Southwest China [8]. This small mammal is similar in appearance to the squirrel and feeds on insects, fruits and small vertebrates. There are six subspecies of *Tupaia belangeri* in China, namely, *T*. *belangeri gaoligongensis*, *T*. *belangeri modesta*, *T*. *belangeri yaoshanensis*, *T*. *belangeri tonquinia*, *T*. *belangeri yunalis* and *T*. *belangeri chinensis* [9]. Previous studies have indicated that tree shrews are closely related to humans in genomic signature, biochemical metabolism and physiological function [10–11]. Due to its small body size, life span, short reproductive cycle and low cost of maintenance, tree shrews have been increasingly used as alternative laboratory animals in recent years. Several human diseases have been successfully studied by using this animal, such as hepatitis, influenza, dengue fever, Alzheimer’s disease, social stress and depression [12–15]. Our previous study [16] revealed the gut microbial community between tree shrew and human was different, if we used tree shrew as laboratory animal to study human diseases, especially in gastrointestinal tract, could this animal reflected the pathophysiological features of human infection? Whether there was somewhat similar gut bacterial populations between tree shrew and human, and this might be helpful to know for future clinical research applications. Because *Proteus* spp. was the most isolated bacteria from feces of tree shrew by using culture method [16], in this study, the complete genome sequencing method was used to analyse the characteristics of *Proteus* spp. isolated from tree shrews, and comparative genomic analysis was performed to reveal their relationships.

## Materials and methods

### Sample collections

Thirty-six tree shrew faecal samples were collected at the Center of Tree Shrew Germplasm Resources, Institute of Medical Biology, Chinese Academy of Medical Science and Peking Union Medical College in Kunming, China. The tree shrews were a closed population and healthy, without visible signs of disease or tumours; 20 were male, and 16 were female. The average age was 36.08±23.04 months, ranging from 2 months to 75 months. All tree shrews were divided into four age groups based on the method of a previous study with some modification [17]. The infant group was defined as under 7 months, the young group was between 8 and 18 months, the middle group was 19 to 42 months, and the senile group was over 43 months. Tree shrews were housed in independent sterilized stainless steel cage containing hygienic food and water without any outside contact. The commercial full-price nutritive pellet was used for feeding twice a day, and the clean apple was fed once a week. The details of the tree shrew information were shown in Table 1.

**Table 1:** The details of tree shrew information and isolation results.

Strain ID	Species	Host sex (tree shrew)	Host age (Months)	Host age group	Host generation
YNTSP1	*P*. *mirabilis*	Male	45	Senile	F4
YNTSP12	*P*. *mirabilis*	Male	14	Young	F4
YNTSP13	*P*. *mirabilis*	Male	15	Young	F4
YNTSP16	*P*. *mirabilis*	Female	27	Middle	F4
YNTSP17	*P*. *mirabilis*	Male	45	Senile	F4
YNTSP19	*P*. *mirabilis*	Female	26	Middle	F4
YNTSP21	*P*. *mirabilis*	Female	26	Middle	F4
YNTSP22	*P*. *mirabilis*	Male	9	Young	F4
YNTSP24	*P*. *mirabilis*	Male	9	Young	F4
YNTSP26	*P*. *mirabilis*	Female	9	Young	F4
YNTSP3	*P*. *mirabilis*	Male	59	Senile	F4
YNTSP34	*P*. *mirabilis*	Female	3	Infant	F4
YNTSP35	*P*. *vulgaris*	Female	2	Infant	F4
YNTSP36	*P*. *mirabilis*	Female	2	Infant	F4
YNTSP40	*P*. *mirabilis*	Male	2	Infant	F4
YNTSP42	*P*. *mirabilis*	Male	2	Infant	F4
YNTSP43	*P*. *mirabilis*	Male	49	Senile	F4
YNTSP44	*P*. *mirabilis*	Female	37	Middle	F4
YNTSP48	*P*. *mirabilis*	Female	62	Senile	F4
YNTSP5	*P*. *mirabilis*	Male	41	Middle	F4
YNTSP53	*P*. *mirabilis*	Male	71	Senile	F4
YNTSP56	*P*. *mirabilis*	Female	56	Senile	F4
YNTSP57	*P*. *mirabilis*	Male	50	Senile	F4
YNTSP58	*P*. *mirabilis*	Female	50	Senile	F4
YNTSP59	*P*. *mirabilis*	Male	52	Senile	F4
YNTSP6	*P*. *mirabilis*	Female	64	Senile	F4
YNTSP62	*P*. *mirabilis*	Male	75	Senile	F4
YNTSP63	*P*. *mirabilis*	Male	58	Senile	F4
YNTSP65	*P*. *mirabilis*	Male	70	Senile	F4
YNTSP66	*P*. *mirabilis*	Female	37	Middle	F4
YNTSP67	*P*. *mirabilis*	Female	37	Middle	F4
YNTSP69	*P*. *mirabilis*	Male	34	Middle	F4
YNTSP70	*P*. *mirabilis*	Male	37	Middle	F4
YNTSP72	*P*. *vulgaris*	Female	74	Senile	F4
YNTSP8	*P*. *mirabilis*	Female	26	Middle	F4
YNTSP9	*P*. *mirabilis*	Male	24	Middle	F4

### Bacterial isolation and DNA extraction

The *Proteus* spp. bacteria were isolated according to previous study [18]. All faecal samples were inoculated on MacConkey Agar and Xylose Lysine Deoxycholate (XLD) agar (Luqiao, Beijing) and incubated at 37°C for 24 hours. All suspected *Proteus* spp. colonies were selected, purified by inoculation on Brain Heart Agar (BHI) (Luqiao, Beijing) at 37°C for 24 hours, and finally identified using the Vitek Compact 2 biochemical identification system (bioMérieux). Total genomic DNA of the isolated bacteria was extracted using a bacterial total genomic DNA extraction kit (Tiangen, Beijing) following the manufacturer’s instructions. All DNA samples were stored at -20°C for complete genome analysis.

### Genome sequencing, assembly and annotation

Bacterial genome sequencing of all isolates was performed by our laboratory on the Illumina MiSeq platform using 2×250 bp paired-end reads. The libraries were built using a Nextera XT DNA Library Prep Kit following the manufacturer’s reference guidelines. Generally, 1 ng genomic DNA of each strain was used. After segment and purification, index PCR was performed to add the Illumina Nextera barcodes using i5 and i7 primers, and then the purification was executed again to remove non-target fragments. Finally, the libraries were normalized, pooled and sequenced using an Illumina MiSeq sequencing system (Illumina, San Diego, USA).

The raw data were trimmed for quality control, and low-quality (<Q40) reads were filtered by Trimmomatic (version 0.38) [19]. Draft genomes were assembled using SOAPdenovo (version 2.04), with k-mer values (25, 31, 37, 47, 59, 71, 83 and 95) optimized to the best assembly results [20–21]. Gapcloser (version 1.12r6) was used to fill the genomic gaps. GenemarkS software (version 4.28) [22] with default parameters was used to predict the open reading frame (ORF) of each genome, and the predicted amino acid sequences were aligned and annotated by DIAMOND (E-value: 1e-5, top 5) [23] to NCBI non-redundant nucleotide database (NR), SwissProt, eggNOG, KEGG, Pfam, Pathogen-Host Interaction database (PHI), Antibiotic Resistance Genes Database (ARDB), and Virulence Factor Database (VFDB). The heatmaps based on PHI, ARDB and VFDB database annotations were drawn using pheatmap in the R package (version 3.2).

### CRISPR, prophage and ICE analyses

Clustered regularly interspaced short palindromic repeats (CRISPR) locus screening of bacteria was predicted by CRT (version 1.2), with minRL 19, maxRL 38, minSL 19 and maxSL 48 [24]. The repeat and spacer sequences of the CRISPR loci for each strain were counted and extracted. The prophage of isolated *Proteus* spp. was analysed by PhiSpy (version 2.3) with default parameters [25]. The integrative and conjugative elements (ICEs) of bacteria were detected by ICEfinder [26], and the sequences of all predicted ICEs were extracted for further analysis.

### Variant analysis

For the detection of single nucleotide polymorphism (SNP) variants relative to the reference, we used clean reads mapped to each reference genome (*Proteus mirabilis* HI4320: NC_010554 and *Proteus vulgaris* FDAARGOS_366: NZ_CP023965) with the BWA software package [27–28]. The mapping quality lower than 20 and sequence depths lower than 5 of the variants were filtered for variants calling [29–30]. The bam files were transferred to sam files using SAMtools, and generated the sequence depths (average = 19.47), coverage (10× = 75.38%) and mapping rate (average = 80.14%). Statistical analyses of SNPs, insertions and deletions (InDels) and classification information for each strain were performed by using BCFtools. The variants of bacteria were annotated based on each reference genome using VCFtools (version 4.2) [31].

### Phylogenetic analysis and statistics

The sequences of the CRISPR repeats were aligned by MEGA 6.0 using the neighbour-joining (NJ) method with 1000 bootstrap replicates to generate a phylogenetic tree. The SNPs of predicted ICEs were called by using the methods mentioned above, and a phylogenetic tree was built by MEGA 6.0 with 18 reference ICE sequences (S1 Table). Phylogenetic analysis based on total SNPs was performed for all isolated *Proteus* spp. in this study with 114 reference *Proteus* spp. (102 *P*. *mirabilis* and 12 *P*. *vulgaris*) from different sources and countries (S2 Table). A maximum-likelihood (ML) tree was generated by applying PhyML version 3.1 with default parameter (data type: nucleotide, the numbers of bootstrap replicates: 100, model: HKY85, gamma value: e, number of relative substitution rate categories: 4 and transition/tranversion rate: e) to the SNPs differentiating the genomes [32]. The distance-based NJ method was also used to estimate their phylogenetic relations. The phylogenetic tree was visualized and ordered with FigTree (version 1.4.3) and iTol.

Statistical analysis was performed using the SPSS software package (version 16.0, IBM, USA). Kolmogorov-Smirnov Z, T-test or Kruskal-Wallis H test were used if appropriate. A P value of <0.05 was recognized as statistically significant. The Tajima’s D values were calculated by MEGA 6.0 for *Proteus* spp. in this study.

The sequence data have been deposited into the European Nucleotide Archive, www.ebi.ac.uk/ena with Accession Numbers ERS3013925 to ERS3013960 (PRJEB30582). The sequences of predicted ICEs were deposited into the NCBI database with GenBank Accession Numbers from MK460213 to MK460220.

## Results

Thirty-four *P*. *mirabilis* and two *P*. *vulgaris* strains were isolated in this study, and the details of the strains with host information were shown in Table 1. For the genomic sequencing results, 12,278,826 raw reads were obtained for 36 *Proteus* spp. bacteria, and 11,608,128 clean reads were retained after quality control. The average clean Q20 (%) was 91.25±0.99, clean Q30 (%) was 88.35±1.28, and effective rate (%) was 93.53±2.73, as shown in Table 2. The average scaffold number (>500 bp) was 1,241.53±174.37, with a range from 890 to 1,698. The minimum scaffold length was 3,358,482 bp, and 3,878,645 bp was the maximum length. The average scaffold GC% content was 39.94±0.25 (Table 2). The predicted gene results showed that the average genome size was 3.6±0.1 Mbp for 36 isolates, with a range from 3.4 to 3.9 Mbp. The gene numbers were 3,741.39±96.94, with a minimum of 3,467 and a maximum of 3,947. The proportion of total gene length accounted for 84.05±0.78 in the genome, as shown in Table 2. The Kolmogorov-Smirnov Z test showed no statistical significance (P>0.05) for all these features. The details for quality control, assembly and gene prediction results of each strain were shown in S3 Table.

**Table 2:** The statistics of all sequencing strains for the quality control, assembly, prediction and mapping results.

Features		Numbers	Mean	Std. Deviation	Minimum	Maximum
Quality control	RawData (reads)	36	341,000	51,965.37	241,964	487,903
	CleanData (reads)	36	322,000	52,110.64	224,518	472,403
	CleanQ20 (%)	36	91.2533	0.99364	89.12	92.97
	CleanQ30 (%)	36	88.3464	1.27638	85.6	90.62
	CleanGC (%)	36	41.2531	0.24703	40.67	41.86
	Effective Rate (%)	36	93.5264	2.72804	83.28	96.54
Assembly	Scaffold total_num (>500 bp)	36	1,241.53	174.372	890	1,698
	Scaffold total_len (>500 bp)	36	3,620,000	104,134.82	3,358,482	3,878,645
	Scaffold N50	36	4,934.86	1,058.41	2,783	8,196
	Scaffold N90	36	1,277.64	210.349	895	2,022
	Max Scaffold (bp)	36	31,000	7,471.51	13,766	50,230
	Min Scaffold (bp)	36	501.64	1.199	501	506
	Scaffold Sequence GC%	36	39.94131973	0.247879671	39.2344172	40.6334767
Gene predict	Genome_size (bp)	36	3,620,000	104,134.82	3,358,482	3,878,645
	Gene_total_length (bp)	36	3,040,000	104,828.49	2,787,918	3,291,156
	Gene_Number	36	3,741.39	96.938	3,467	3,947
	Gene_average_length (bp)	36	813.36	29.04	720	859
	Gene_total_length/Genome (%)	36	84.0486	0.77641	81.36	85.03
Mapping to references	Avg_depth	36	19.47	3.29	16.53	24.95
	Coverage> = 1× (%)	36	88.83	1.27	86.82	89.93
	Coverage> = 4× (%)	36	84.77	1.72	81.82	86.2
	Coverage> = 10× (%)	36	75.38	3.27	70.59	79.03
	Map_rate (%)	36	80.14	7.78	67.58	88.99

**Fig 1:**
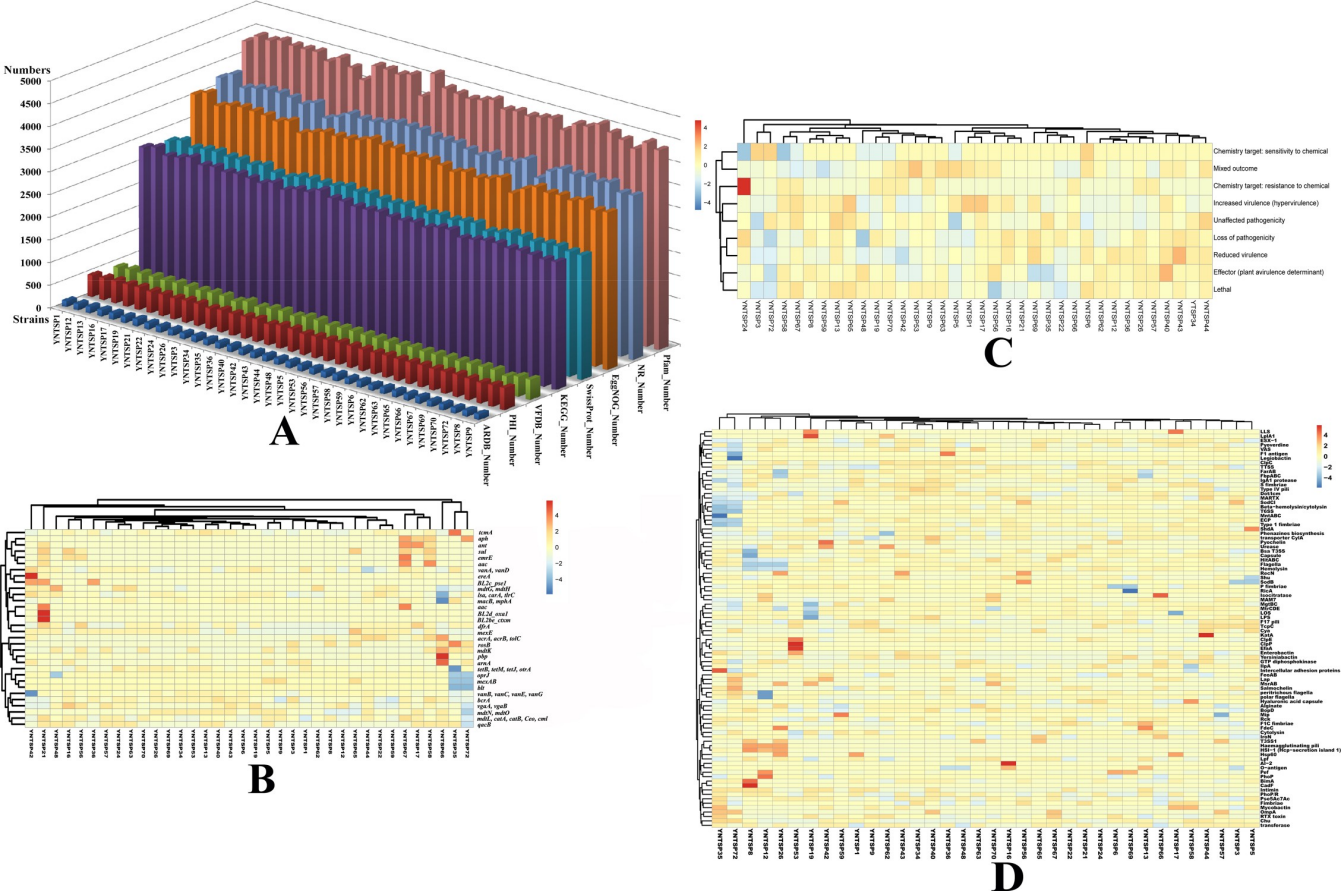
The annotation results of *Proteus* spp. bacteria in this study. A. The number of annotated genes of each strain based on Pfam, NR, EggNOG, SwissProt, KEGG, PHI, VFDB and ARDB databases. B. The heatmap of annotated ARDB resistance genes for each isolate. C. The heatmap of PHI database annotation results. D. The heatmap of annotated virulence factors based on the VFDB database.

In total, 132,584 NR, 99,769 SwissProt, 159,615 Pfam, 101,954 KEGG, and 126,679 eggNOG numbers were annotated with average numbers 3682.89±90.37, 2771.36±36.01, 4433.75±113.67, 2832.06±42.49, and 3518.86±74.82, respectively (Fig 1A). A total of 4,803 genes were annotated in the ARDB database for all *Proteus* spp. in this study, with an average number of 133.42±6.31 and a range from 120 to 151 (Fig 1A). We found 24.46% of isolates carried *macB* and *mphA* genes, which were associated with resistance to macrolide; 10.04% of isolates carried *vanB*, *vanC*, *vanE* and *vanG* genes, which were resistant to vancomycin; 8.59% of strains carried *lsa*, *carA* and *tlrC* genes, which were resistant to lincosamide, streptogramin, and macrolide; 8.40% of isolates had *bcrA* gene with bacitracin resistance profile; 7.70% of strains possessed *vanA* and *vanD* genes, which were resistant to vancomycin and teicoplanin; 6.25% of isolates carried *tetB*, *tetM*, *tetJ* and *otrA* genes, which were associated with resistance to tetracycline; 5.77% of strains had *mdtL*, *catA*, *catB*, *ceo* and *cml* genes, which referred to multidrug resistant genes; 4.84% of isolates had *pbp* gene, which indicated the penicillin resistance. All those ARDB annotated genes and profiles mentioned above were shown in hot spots of Fig 1B. There were 17,185 PHI annotated genes for all strains; the average number was 477.36±12.52, with a range from 449 to 503 (Fig 1A). Reduced virulence (53.28%), unaffected pathogenicity (15.87%), loss of pathogenicity (13.24%), and increased virulence (hypervirulence) (5.43%) were the dominant PHI phenotypes of the mutant deposited in the PHI database, as shown in Fig 1C. The virulence factor investigation showed 15,577 annotated genes obtained from the VFDB database in total, and the average number was 432.69±18.87, with a range from 377 to 470. Flagella (9.24%) was the most annotated virulence factor overall, followed by LOS (lipooligosaccharide) (7.27%), type 1 fimbriae (4.21%), peritrichous flagella (3.96%), type IV pili (3.48%), HitABC (3.28%), capsule (3.07%), P fimbriae (3.03%), FbpABC (2.94%), T6SS (2.89%), pyoverdine (2.28%), alginate (2.02%), and TTSS (2.00%) (Fig 1D). Two isolated *P*. *vulgaris* strains had different virulence factors than *P*. *mirabilis*; for example, a high proportion of ompA and RTX toxins were found in *P*. *vulgaris*, but a low proportion of T6SS and type 1 fimbriae were found, as shown in Fig 1D. The annotation numbers according to each database had no statistical significance (P>0.05) among the isolates.

**Fig 2:**
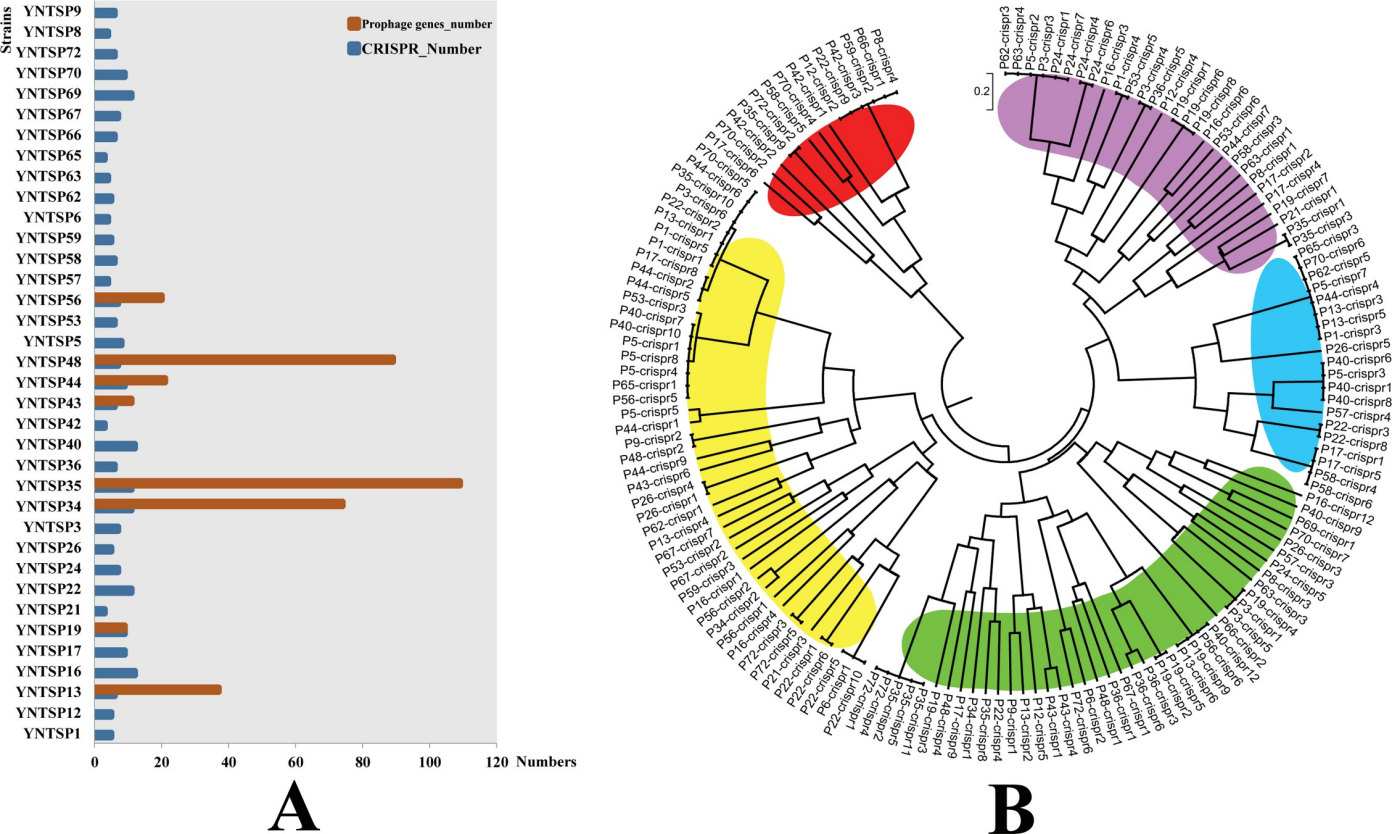
The CRISPR and prophage results in this study. A. Statistical analysis of the numbers of CRISPR loci and prophage genes in each strain. B. Phylogenetic tree according to sequences of the CRISPR repeats.

CRISPR analysis revealed 245 loci for all strains. Among them, YNTSP16 and YNTSP40 had 12 CRISPR loci, followed by YNTSP22 (11 loci), YNTSP34 (11 loci), YNTSP35 (11 loci), and YNTSP69 (11 loci) (Fig 2A). The details of CRISPR information for each strain were shown in S4 Table. The phylogenetic analysis based on sequences of CRISPR repeats showed five clustering groups for all *Proteus* spp. bacteria, presented with red, yellow, green, blue and purple colours, as shown in Fig 2B. The sequences of the CRISPR repeats revealed highly polymorphic characteristics of the isolates in this study, since few identical sequences were found in the phylogenetic tree. However, only eight strains (YNTSP13, YNTSP19, YNTSP34, YNTSP35, YNTSP43, YNTSP44, YNTSP48 and YNTSP56) had prophages, as shown in Fig 2A. The largest numbers of prophage genes were found in YNTSP35 (109), followed by YNTSP48 (89), and YNTSP34 (74); the annotation details of all prophages were shown in S5 Table.

**Fig 3:**
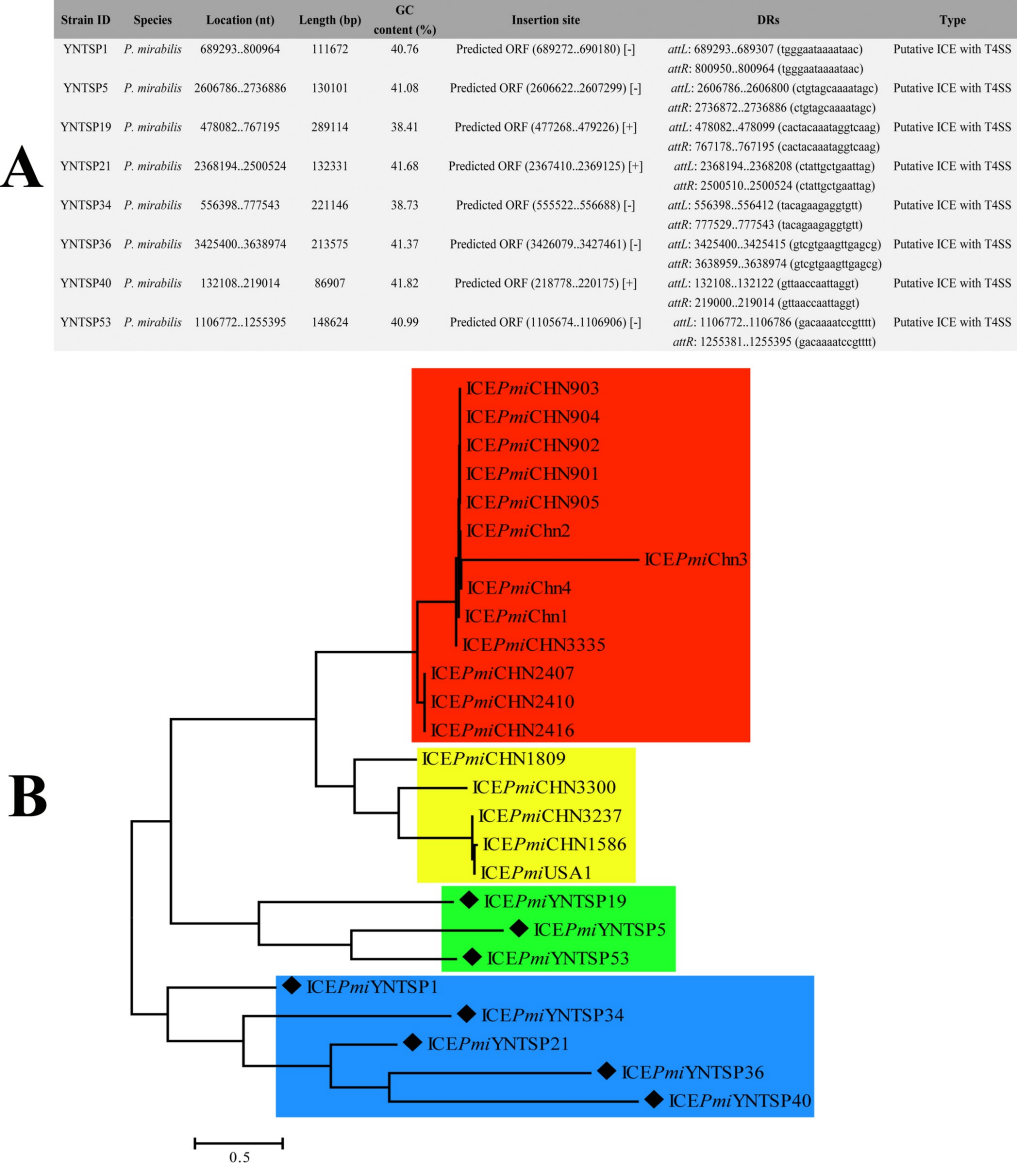
ICE analysis results in this study. A. The details of eight predicted ICEs in this study. B. Phylogenetic tree based on total SNPs of eight ICEs from *Proteus* spp. with references.

The ICE prediction results showed that eight *P*. *mirabilis* isolates had putative ICEs with T4SS structures and belonged to SXT/R391 family, as shown in Fig 3A. Although all predicted ICE types (SXT/R391) were identical, high diversity was found among them. The locations and insertion sites of ICEs were different. The largest length ICE was 289,114 bp (ICE*Pmi*YNTSP19), while the shortest was 86,907 (ICE*Pmi*YNTSP40). Phylogenetic analysis revealed four clustered groups, indicated with red, yellow, green and blue colours (Fig 3B). Eight ICEs in this study were clustered in green and blue groups, differentiated with other reference ICEs (red and yellow groups). All of the reference ICE strains were isolated from diarrhoea patients or domestic animals (S1 Table), while all of the isolates in this study were from laboratory animals.

**Fig 4:**
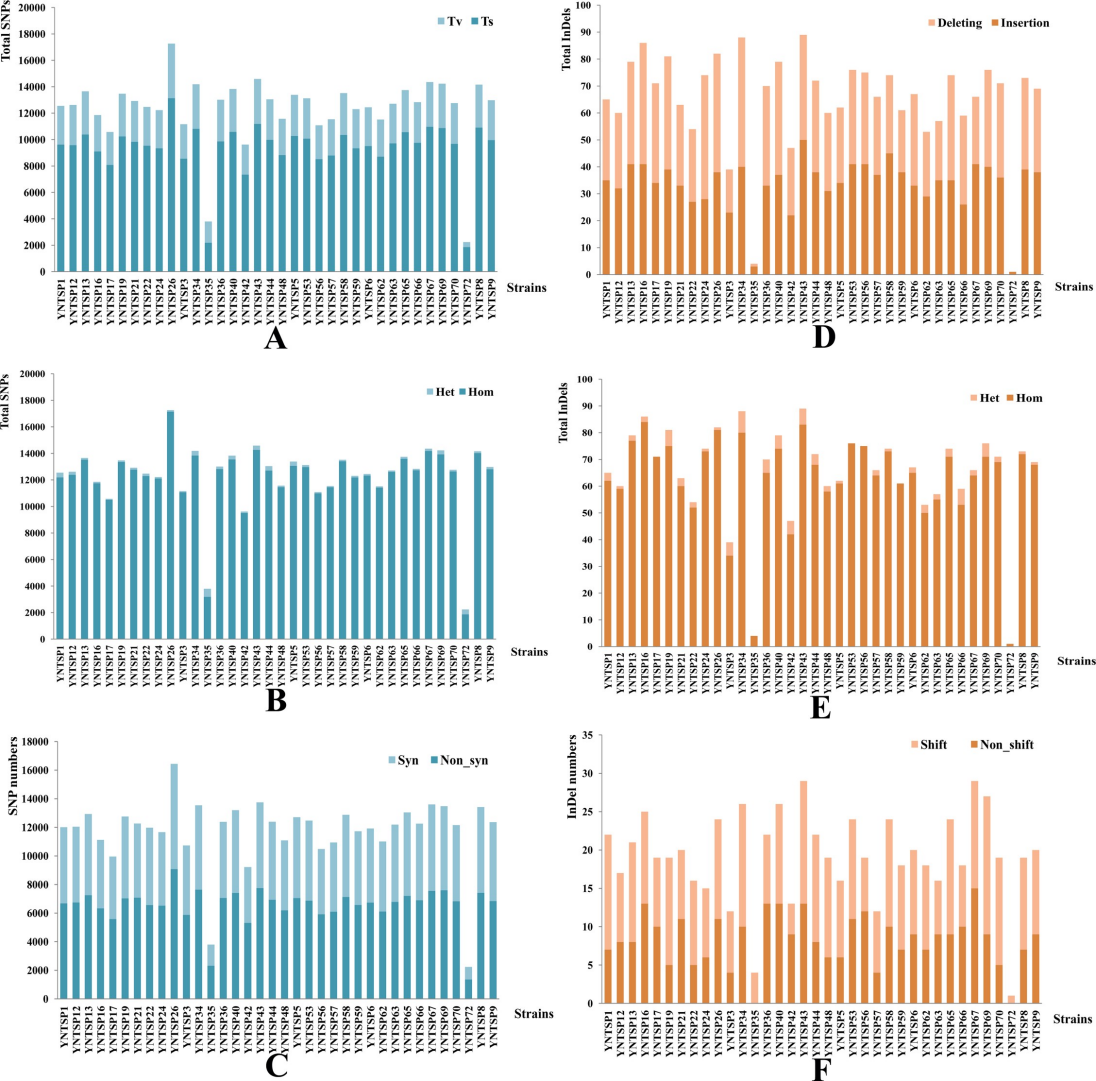
SNP variants and InDel analysis in this study. A. Comparison of the base transitions (Ts) and base transversions (Tv) for SNPs. B. Comparison of the homozygous mutations (Hom) and heterozygous mutations (Het) for SNPs. C. Comparison of the synonymous mutations (Syn) and non-synonymous mutations (Non-Syn) of SNPs. D. Comparison of the insertions and deletions for InDels. E. Comparison of the homozygous mutations (Hom) and heterozygous mutations (Het) for InDels. F. Comparison of the non-frame shift (Non-shift) and frame shift (Shift) for InDels.

For SNP variant investigation, *P*. *mirabilis* HI4320 and *P*. *vulgaris* FDAARGOS_366 were used as reference genomes. Thirty-four *P*. *mirabilis* and two *P*. *vulgaris* strains in this study were mapped to each reference. The depths for all of the sequencing results were >16, with an average depth of 19.47±3.29, and the coverage of sequencing was shown in Table 2. The average mapping rate for all of the *Proteus* spp. was 80.14±7.78. In total, 422,163 SNPs were identified for all of the *Proteus* spp., with an average of 11,700±2,493.98 SNPs. However, only 3,794 and 2,234 SNPs were found for two *P*. *vulgaris* (YNTSP35 and YNTSP72). A total of 9320.24±2117.44 base transitions (Ts) were obtained for *Proteus* spp., compared with 2910.35±602.98 base transversions (Tv) (Z = 4.007, P = 0.000), as shown in Fig 4A. The number of homozygous mutations (Hom) was much higher than the number of heterozygous mutations (Het) in this study (Z = 4.243, P = 0.000), and the average number was 12,014.50±2,857.33 and 214.29±262.21, respectively (Fig 4B). Statistical significance was also found between synonymous mutations (Syn) and non-synonymous mutations (Non-Syn) (Z = 3.517, P = 0.000), showing 5,397.29±599.89 and 6,841.97±709.67 mutations, respectively (Fig 4C). There were 695 InDels for all *Proteus* spp. with an average of 19.31±6.00 InDels. In general, 33.72±9.76 were insertions, and 31.36±10.51 were deletions. No significant difference was found between the insertions and deletions (Z = 0.946, P = 0.336) (Fig 4D). A large number of Hom were identified compared with Het (Z = 4.007, P = 0.000), and the average number was 62.53±18.45 for Hom and 2.56±2.12 for Hem (Fig 4E). The average number for non-frame shift (Non-shift) InDels was 8.42±3.19, compared with 10.89±3.96 for frame shift (Shift), as shown in Fig 4F. Statistical significance was found between non-shift and shift InDels (Z = 1.532, P = 0.018).

**Fig 5:**
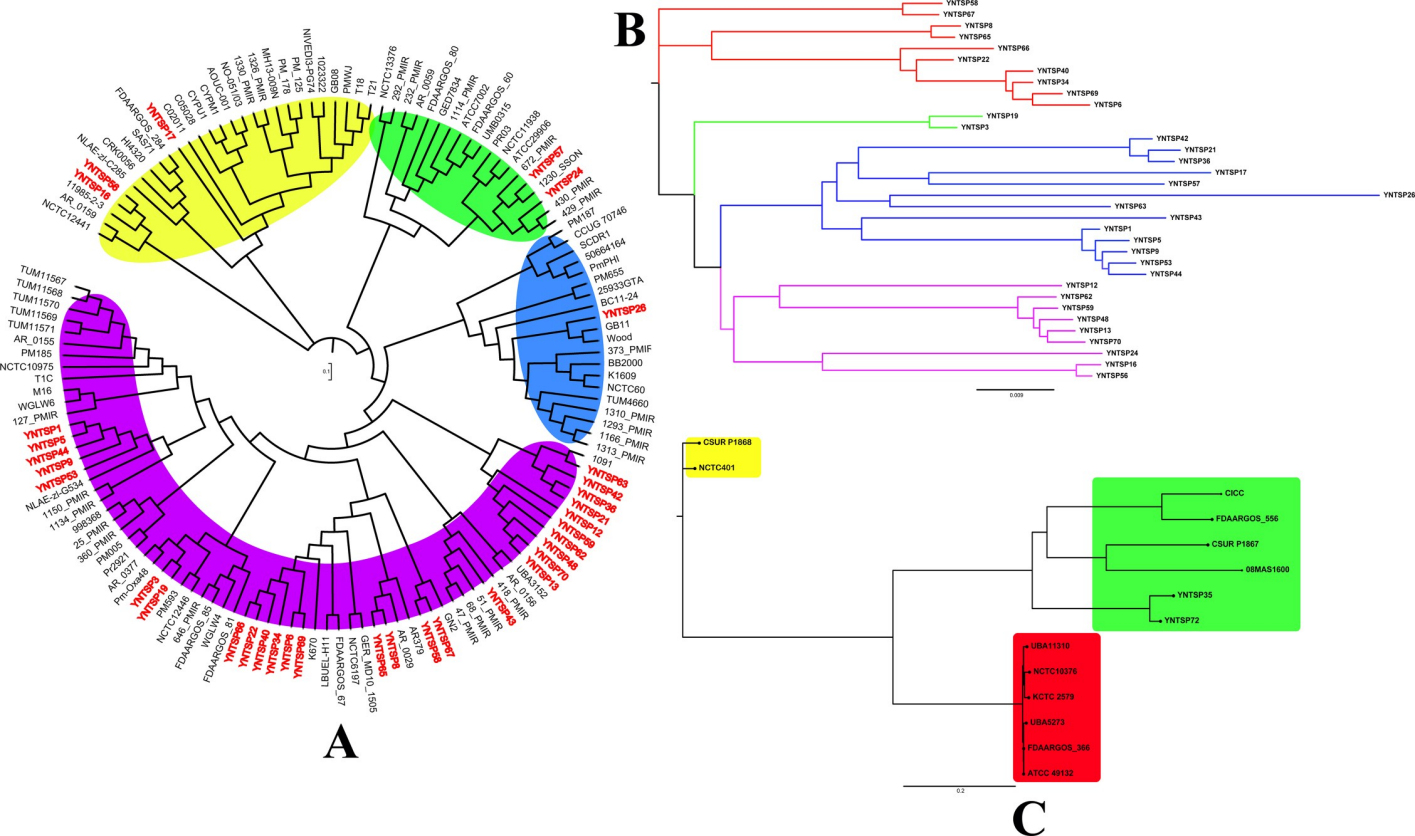
Phylogenetic analysis based on ML method of *Proteus* spp. strains in this study. A. Phylogenetic tree of 34 *P*. *mirabilis* isolated from tree shrews with 102 reference strains. B. Phylogenetic tree of 34 *P*. *mirabilis* isolated from tree shrews in this study. C. Phylogenetic tree of *P*. *vulgaris* in this study.

**Fig 6:**
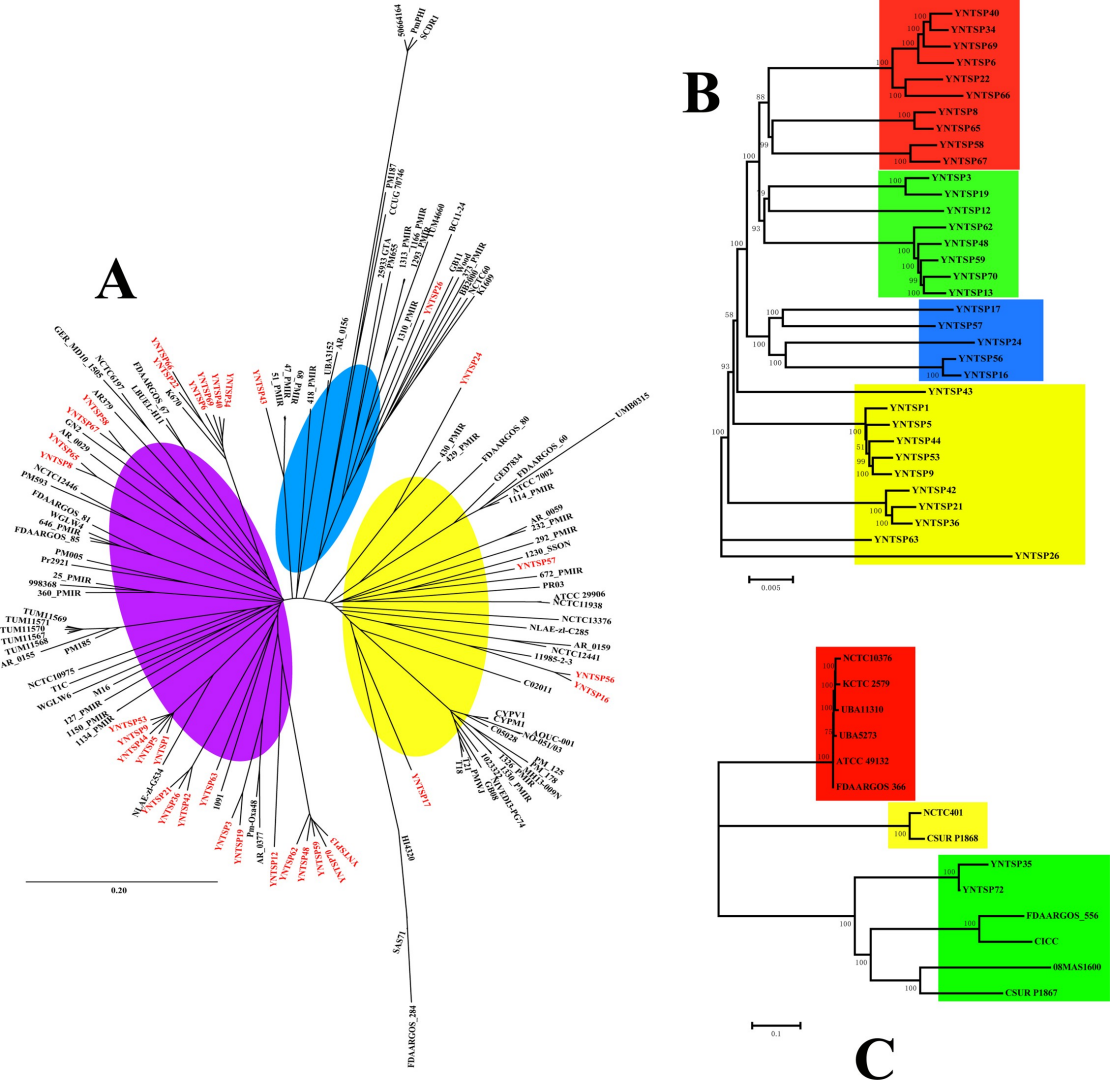
Phylogenetic analysis based on NJ method of *Proteus* spp. strains in this study. A. Phylogenetic tree of 34 *P*. *mirabilis* isolated from tree shrews with 102 reference strains. B. Phylogenetic tree of 34 *P*. *mirabilis* isolated from tree shrews in this study. C. Phylogenetic tree of *P*. *vulgaris* in this study.

Phylogenetic analysis based on ML method of *P*. *mirabilis* indicated high genetic diversity for strains isolated from tree shrews because 136 isolates were divided into four clustering groups, indicated with yellow, green, blue and purple colours (Fig 5A), and each group had *P*. *mirabilis* bacteria isolated in this study (red font). Twenty-eight strains were clustered into the purple group. Although ten isolates (YNTSP63, 42, 36, 21, 12, 59, 62, 48, 70, and 13) were located at an identical branch, others were clustered into different branches of the tree. Most of the reference strains in the purple group were isolated from humans, especially from urinary tract infections, and *P*. *mirabilis* from tree shrews showed high similarity with these strains, such as 1150_PMIR, 1134_PMIR, Pm_Oxa48, K670, GN2, 51_PMIR, 418_PMIR and 1091. Furthermore, these reference strains were isolated from different countries without any epidemiological relationship. YNTSP26 was clustered into the blue group, and most strains in this group were also isolated from patients, except *P*. *mirabilis* Wood, which was isolated from *Lucilia sericata*, BC11-24 from swine, and 25933GTA from beef. There were two strains (YNTSP57 and YNTSP24) divided into the green group, and all of the reference isolates were isolated from patients. The last three *P*. *mirabilis* bacteria from tree shrews were clustered into the yellow group, and the major sources of strains were from patients as well, except for 11985-2-3 and SAS71, which were environmental strains. We further performed phylogenetic analysis of *P*. *mirabilis* isolated from tree shrews independently, and 34 strains were clustered into four groups, as shown in Fig 5B. The four groups were presented with red, green, blue and pink coloured branches. Thirteen strains were clustered into the blue-branch group, followed by ten strains in red and nine in pink; only two isolates were in the green group (Fig 5B). There was no obvious correlation between clustering groups and host information, such as sex, age or age groups. The sex and age groups of tree shrews were randomly distributed in each clustering group. Fourteen *P*. *vulgaris* were separated into three groups, including two strains isolated from tree shrews in this study and 12 references (Fig 5C). YNTSP35 and YNTSP72 were clustered into the green group, showing close similarity with 08MAS1600, CSUR P1867, FDAARGOS_556 and CICC. These reference strains were primarily isolated from the environment or from humans. Other reference isolates were clustered into the yellow and red groups. The alignments and tree files mentioned above were shown as S1 Appendix. The distance-based NJ method for phylogenetic analysis revealed similar results with ML method. Generally, three clustering groups were generated by NJ method, shown with purple, yellow and blue colours (Fig 6A). The clustering results of strains for each group showed close similarity with ML method. Most of the *P*. *mirabilis* from tree shrews were clustered into purple group; YNTSP26 and YNTSP43 were divided into blue group, and the rest of strains were clustered into yellow group (red font). Phylogenetic analysis of 34 *P*. *mirabilis* isolated from tree shrews demonstrated four clustering groups were obtained by NJ method, shown with red, green, blue and yellow colours (Fig 6B). In addition, three clustering groups were generated for *P*. *vulgaris* by NJ method, and the grouping results were identical with ML method mentioned above (Fig 6C). All the alignments and tree files were shown as S2 Appendix by NJ method. In general, the host specificity of *Proteus* spp. bacteria was not discovered, especially for *P*. *mirabilis*. The Tajima’s D values indicated that no statistical significance were found for 34 *P*. *mirabilis* isolated from tree shrews and *P*. *vulgaris* (D = -0.076, P = 0.940; D = 0.742, P = 0.458). However, statistical significance (D = -2.107, P = 0.035) was identified for all 136 *P*. *mirabilis* in this study.

## Discussion

A previous study showed that many wild and domestic animals are hosts of *Proteus* spp., including mammals, birds, amphibians, reptiles, and insects [5]. These bacteria may play a fundamental role in animal pathogenic or physiological microbiota, especially in gastrointestinal tracts. For example, Bittar et al. [33] identified both *P*. *mirabilis* and *P*. *vulgaris* inhabited in the intestines of western lowland gorillas. Gaastra et al. [34] isolated *P*. *mirabilis* from the faeces and urine of dogs with urinary tract infections, and Kroemer et al. [35] reported that *P*. *mirabilis* bacteria caused urinary tract infections in dogs and cats in European countries. Lowe et al. [36] revealed that *P*. *mirabilis* or *P*. *vulgaris* strains dominated the pig tonsil, and Kobashi et al. [37] identified *P*. *mirabilis* from pig faecal samples that was resistant to tetracycline. Other authors emphasized the role of wild birds in the transmission and spread of *Proteus* spp. bacteria to domestic poultry, cattle, or humans [38–39]. However, there is no systemic report about the relations between *Proteus* spp. with laboratory animals, specifically for the tree shrew. Our results indicated that *Proteus* spp. strains were the most commonly isolated bacteria from the gut of tree shrews, even though all of these laboratory animals were closed populations without any contact from outside environments.

*Proteus* species possess different antibiotic resistant abilities. Previous studies on clinical patients revealed that polymyxins resistance was mediated via lipid A of bacteria, and they also had intrinsic resistant abilities to tetracycline, tigecycline and colistin [40–41]. Whole genomic sequencing of *P*. *mirabilis* HI4320 reference strain demonstrated *tetAJ* encoded tetracycline resistance, *cat* encoded chloramphenicol acetyltransferase, *bcr* encoded sulfonamide resistance, and multidrug efflux genes *mdtG*, *mdtH*, *mdtK*, *mdtL* were all contained in this uropathogenic strain [42]. Recently, *P*. *mirabilis* acquired *Salmonella* genomic island 1 (SGI1) was reported, and it was resistant to tetracycline, sulfonamides, fluoroquinolones, streptomycin, trimethoprim, chloramphenicol, and β-lactam antibiotics [43]. Furthermore, *P*. *mirabilis* strain PM58, an extensively drug-resistant isolate contained the New Delhi metallo-β-lactamase 1 (*NDM-1*) gene was also reported. *P*. *mirabilis* isolate (NO-051/03), a recent sequenced strain from a patient acquired β-lactam, trimethoprim, sulfonamides, and aminoglycosides resistant genes [44]. Compared the ARDB annotation results of tree shrew *Proteus* spp. strains with these clinical ones, we found similar antimicrobial resistant genes acquired by both *Proteus* spp. isolates. In our study, ARDB annotations indicated high proportions of macrolide resistance profile. The tetracycline resistance profiles were involved in *tetB*, *tetM*, *tetJ* and *otrA* genes, and the descriptions of those genes were tetracycline efflux pump and ribosomal protection protein. *mdtL* and *cat* genes with chloramphenicol resistance profile were annotated, and they mainly referred to multidrug resistance efflux pump and chloramphenicol acetyltransferase. *lsa*, *carA* and *tlrC* genes annotated with lincosamide, streptogramin, macrolide resistance profile. The penicillin resistance profile with *pbp* gene was also annotated. In addition, our previous study [16] revealed that some of the *Proteus* spp. strains possessed *NDM-1* gene resistant to Imipenem. All these pieces of evidences indicated that the antimicrobial resistant profiles or genes were similar between tree shrew *Proteus* spp. isolates with human clinical ones.

The evolutionary relationships of different niches in humans, animals, and plants using proper techniques could reveal the roles of *Proteus* spp. bacteria in these hosts. It would be interesting to compare the features of *Proteus* spp. from clinical isolates and animals, explaining the factors contributing to the different lifestyles of *Proteus* spp. in various environments [5]. *Proteus* species are considered potential pathogenic bacteria in human gastrointestinal or urinary tract infections [45–46]. It is postulated that human guts are the reservoir of *Proteus* spp., especially for the *P*. *mirabilis* species. However, the interactions between *Proteus* spp. and hosts may lead to the pathogenicity of this genus resulting from population expansion. Several virulence factors of this genus have been revealed in previous studies. *Proteus* spp. possess similar characteristics to other *Enterobacteriaceae* for lipopolysaccharides (LPS), such as *Salmonella* spp. and *Escherichia coli*. The chemical structure of LPS may lead to the adaptation of *Proteus* spp. bacteria to environmental conditions and enhance their pathogenic abilities [47]. In addition, bacterial flagella are pathogenesis factors of *Proteus* spp. due to their immunogenic structure. The flagellins activate the inflammatory pathways of hosts and contribute to the stimulation of the host innate immune systems [48–49]. Furthermore, one of the essential pathogenesis factors of *Proteus* spp. infections is adhesion to epithelial cells or surfaces for urinary or gastrointestinal tracts. A previous study [50] showed 17 fimbrial operons for *P*. *mirabilis* HI4320, and six types of fimbriae have been characterized thus far, including MR/P fimbriae (mannose-resistant *Proteus*-like fimbriae), MR/K fimbriae (mannose-resistant *Klebsiella*-like fimbriae), NAF (non-agglutinating fimbriae), ATF (ambient-temperature fimbriae), PMP (*P*. *mirabilis* P-like pili), and PMF (*P*. *mirabilis* fimbriae). All of these fimbriae play an important role in the pathogenesis of *P*. *mirabilis* infections. In this study, all of the *Proteus* spp. strains isolated from tree shrews possessed the virulence factors mentioned above, such as LPS, flagella, type 1 fimbriae and P fimbriae. Therefore, the laboratory animal-isolated *Proteus* spp. strains showed no significant differences from clinical strains, especially in their pathogenicity.

Furthermore, phylogenetic analysis based on total SNPs of strains demonstrated that *P*. *mirabilis* from tree shrew had closer genomic relations with human clinical isolates. There was no host specificity of *Proteus* spp. isolated from human patients, animals or environments. Close similarity could be found between strains from different epidemiological resources, and even within the same host species, such as the tree shrews in this study, and characteristics of high genetic diversity in the isolates were also identified. This unexpected result contradicted previous studies, and several studies have indicated that microorganism specificity to their hosts supports the co-evolution of the host and its microbes [51–52]. However, comparative genomics of the *Proteus* spp. strains from our results with other references did not reveal host specificity of isolates.

Based on ARDB, VFDB annotations and phylogenetic relation analysis of tree shrew *Proteus* spp., we found the antimicrobial resistance, virulence factors and genomic features of these isolates were similar with human clinical *Proteus* spp. strains. These results indicated some of the gut bacterial population was somewhat similar between tree shrew and human, specifically for *Proteus* spp. However, the antimicrobial resistant bacteria and related genes, especially for erythromycin, tetracycline and β-lactamase, were commonly existed in this laboratory animal. This should be paid attention in future clinical research applications.

## Conclusions

In this study, we firstly analyzed the genomics of *Proteus spp*. isolated from tree shrews and compared their characteristics with other references. Overall, the host specificity of isolates was not discovered and *Proteus* spp. strains were somewhat similar between tree shrew and human. This investigation provided important information on characteristics of genome for *Proteus* spp. isolated from tree shrews.

## Supporting information

S1 Table The information for reference ICE sequences in this study. (XLS)

S2 Table The information for the 114 reference *Proteus* spp. genomes. (XLS)

S3 Table The details of the quality control, assembly and gene prediction results in this study. (XLS)

S4 Table The details of the CRISPR information for each strain. (XLS)

S5 Table The annotation details of all prophages in this study. (XLS)

S1 Appendix The alignments and tree files of *Proteus* spp. based on ML method in this study. (RAR)

S2 Appendix The alignments and tree files of *Proteus* spp. based on NJ method in this study. (RAR)
